# Interaction Between Chitosan and Mucin: Fundamentals and Applications

**DOI:** 10.3390/biomimetics4020032

**Published:** 2019-04-25

**Authors:** Mar Collado-González, Yadira González Espinosa, Francisco M. Goycoolea

**Affiliations:** School of Food Science & Nutrition, University of Leeds, Leeds LS2 9JT, UK; y.gonzalezespinosa@leeds.ac.uk (Y.G.E.); f.m.goycoolea@leeds.ac.uk (F.M.G.)

**Keywords:** chitosan, mucin, interactions, complexes, mucoadhesion, mucosa

## Abstract

The term chitosan (CS) refers to a family of aminopolysaccharides derived from chitin. Among other properties, CS is nontoxic, mucoadhesive and can be used for load and transport drugs. Given these and other physicochemical and biological properties, CS is an optimal biopolymer for the development of transmucosal drug delivery systems, as well as for the treatment of pathologies related to mucosal dysfunctions. Mucins are glycoprotein macromolecules that are the major components of mucus overlaying epithelia. CS interacts with mucin and adsorbs on and changes the rheology of mucus. However, CS and mucins denote families of polymers/macromolecules with highly variable chemical structure, properties, and behavior. To date, their interactions at the molecular level have not been completely unraveled. Also, the properties of complexes composed of CS and mucin vary as a function of the sources and preparation of the polymers. As a consequence, the mucoadhesion and drug delivery properties of such complexes vary as well. The breadth of this review is on the molecular interactions between CS and mucin. In particular, in vitro and ex vivo characterization methods to investigate both the interactions at play during the formation of CS-mucin complexes, and the advances on the use of CS for transmucosal drug delivery are addressed.

## 1. Introduction

Epithelium comprises all the free surfaces of the body—the skin and the surfaces of internal cavities and tubular organs. The most external layer of the epithelium is called the mucosa. Depending on its location, the mucosa receives different names, i.e., oral, nasal, anal, gut, or intestinal mucosa. The exposure of epithelium to the external environment leads to several risks, such as the loss of water and thus the desiccation of surfaces, or the attack of bacteria, fungi, or viruses. Thus, to protect itself from risks derived from the exposure to environmental conditions or to pathogens, the more external layers of cells synthesize and secrete mucus.

## 2. The Mucus Layer

Mucus is a semipermeable barrier that allows the exchange of water, nutrients, gases, active biomolecules such as hormones and odorants, and cells such as gametes. However, mucus constitutes a barrier for most of the pathogens [1]. In this sense, the mucus layer is a first level defence against the colonisation of the subjacent epithelial cells [2,3].

**Figure 1:**
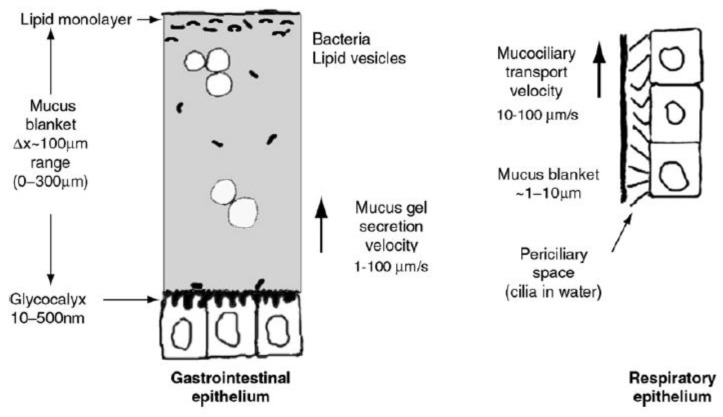
Structure of the mucus gel on the epithelial surfaces. Reprinted from [1], with permission from Elsevier.

The structure of the mucus layer on the epithelial surfaces has different regions. In contact with the subjacent cells through the glycocalyx, there is a layer firmly attached—known as the unstirred layer—which gives lubricity to the mucosal surfaces (Figure 1). The next region is a mucus blanket, whose thickness varies as a function of the epithelium in which it is located [1,4,5]. The furthest region from the cell surface is the lipid monolayer, which is exposed to the free surface [1]. The mucus layer is dynamic. Mucus is continuously synthesized, degraded, shed, and digested. These processes have different rates as a function of the physiological conditions or the action of the microbiota, among other factors [3,4,6,7].

The structure and composition of mucus determine its properties to a large extent. Mucins are the main component of mucus, which are a family of highly glycosylated proteins (>10^6^ Da) [8] synthesised by all cells in the surface mucosa and some gastric glands [9]. The functions in which mucins are involved are the adhesion of the unstirred layer to the cell surface, the formation of a matrix that is a physical barrier in which pathogens and nanomaterials are trapped [1], and the viscoelasticity of mucus, among others. The viscoelasticity of mucus varies as a function of the mucin properties, mucin content, mucus hydration [1,10], and the environmental conditions, due to the electrostatic repulsive forces that appear on the mucins as a result of the negative surface charge of mucins at pH > 2 [11]. Mucus can behave as a viscous fluid, which allows motility of the bacteria and sperm trapped in it, and as an elasticsemisolid, which cannot be removed from mucosal surfaces. An example of the worst case of the latter is in the cystic fibrosis illness [1].

**Figure 2:**
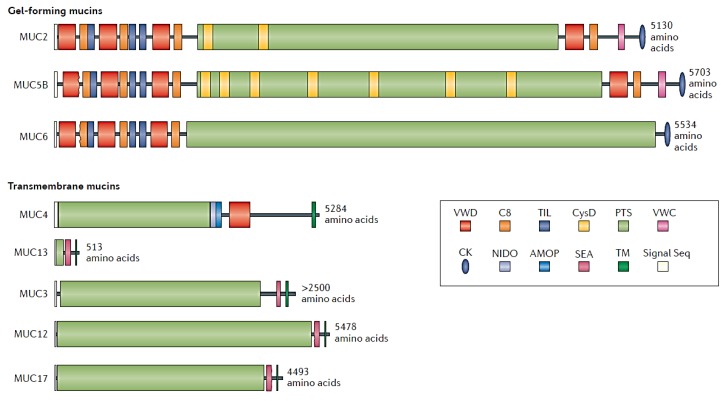
The domain structure of mucins. VWD: Von Wiellebrand D-domain, C8: conserved cysteine rich domain, TIL: trypsin inhibitor-like cystein-rich domain, CysD: domain rich in cystein residues, VWC: Von Wiellebrand C-domain, PTS: PTS domains, CK: cystein knot domain, NIDO: nidogen homology region, AMOP: the adhesion-associated domain in MUC4 and other proteins, SEA: sea-urchin sperm protein, enterokinase, and agrin domain, TM: transmembrane domain. Reprinted with permission from Springer Nature, Copyright 2016 [2].

**Figure 3:**
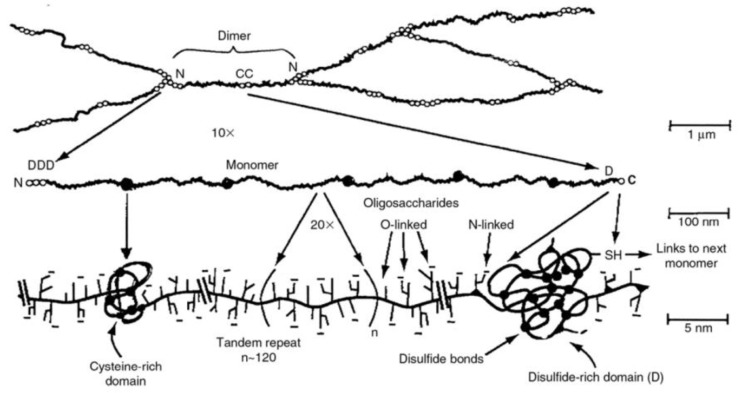
Representation of the crosslinking of mucins. Extracted with permission from [1].

There are at least 15 types of mucins, which are classified as cell-associated mucins and gel-forming mucins depending on their location [2,12].Although cell-associated mucins have a transmembrane domain and an SEA (sea-urchin sperm protein, enterokinase, and agrin) domain [1], all mucins share the same structural features (Figure 2)—along the linear proteins peptide backbone they are highly glycosylated in the central region, while the C-terminal and N-terminal regions remain unglycosylated [13]. Mucins show alternative hydrophilic and hydrophobic regions, which are essential to the gel formation [9]. The hydrophilic regions of mucins present a varied number of PTS (proline (P)–threonine (T)–serine (S)) domains, which are highly O-glycosilated through the T and S residues. The glycan portion accounts for 40–80% of the mass in secreted mucins [4]. The size of the glycans [14], as well as the composition and the pattern of glycosylation, varies among mucins [15,16] and within the same type of mucins [3]. In fact, 1–20 monosaccharides are attached as linear or branched polysaccharides through O-glycosidic linkage to mucins [17]. Some glycans have in their structure sialic acid, which along with the N-linked sulphate groups, confers a negative charge to the mucins at a neutral pH [15,18]. The hydrophobic domains of mucins contain domains similar to the von Willerbrand C and D domains [19,20], which are rich in cysteine and charged aminoacids [2,8]. The hydrophobic regions establish disulphide bonds between them, leading to the formation of a branched network of mucins [8,9] (Figure 3). The electrostatic interactions also contribute to the formation of the mucin gel, due to the interaction between hydrophilic regions [1,8]. By means of high-resolution scattering techniques (synchrotron small-angle X-ray scattering (SAXS), small-angle neutron (SAN), dynamic light scattering (DLS) and static light scattering (SLS)), it has been possible to uncover that mucin has a cylindrical, double-globular (also known as “dumbbell”) comb macromolecular structure [21]. Therefore, the mucin gel is a dynamic structure that can trap particles within it, thanks to the electrostatic bonds that are continuously formed between mucins and with any other charged particles within the mucus. As a result, mucins can form aggregates up to 20 MDa [10].

Mucins have gained increasing traction from the biomedical and biotechnological fields, particularly in drug delivery, biolubrication, and mucoadhesion areas [13]. Due to the ability of mucins to form hydrogels [13] as a function of the environmental conditions, such as pH and ionic strength [8,10,19], these glycoproteins are involved in the absorption of water and nutrients and in the relationships with bacteria, enhancing the colonisation by symbiotic bacteria and avoiding the attachment of pathogenic ones [22]. At a neutral pH, mucins have an isotropic random coil conformation that is changed when the pH is decreased. At pH < 4, the conformation of mucins corresponds to an anisotropic rod-like structure [8,19], and the interactions between hydrophobic regions in mucin macromolecules are facilitated. Moreover, the electrostatic repulsive forces that appear as a consequence of the presence of sialic acids, sulfate groups, and negatively charged residues in the sequence of the mucins are responsible for maintaining the extended conformation of the rod-like structures [10]. Therefore, the gel formation is enhanced.

The administration of drugs through mucosal epithelia (i.e., oral, nasal, anal, pulmonary, ocular, trans vesicular, or vaginal) requires the development of mucoadhesive drug delivery systems. In this context, the studies developed with natural and synthetic polymers (which interact with mucins) [13] help us to understand the mechanism of mucoadhesion and as a result of this enabling the creation of targeted mucosal therapies.

**Figure 4:**
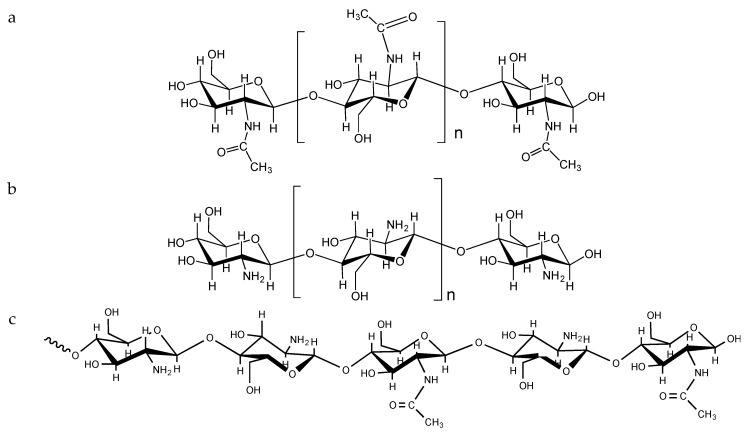
Structure of fully N-acetylated chitin (**a**), fully N-deacetylated chitosan (**b**) and representative partially N-acetylated chitosan sequence (**c**).

Chitin is one of the most abundant polysaccharides in nature, with an estimated annual production of 10 to 100 trillion of metric tons [23,24]. Chitin can be found in the exoskeleton of arthropods, in the fungus of mycelia, and in squid pens, among other sources [25,26]. Chemically, chitin is a linear homopolymer composed of *N*-acetyl-d-glucosamine attached by β(1→4) linkage [27] (Figure 4), which can be found in at least two isoforms according to the orientation of the chains, α- and β-chitin [28,29]. Chitin is a biocompatible polymer, which shows desirable properties in the promotion of wound healing or the suppression of tumorigenic cells [26]. Although it is possible to produce biomaterials from chitin, such as hydrogels or nanofibres [23], the use of chitin is limited due to its low solubility in aqueous systems. To overcome this drawback, chitin can be deacetylated [30,31,32], yielding a family of polymers that show increased solubility in aqueous solvents. These polymers are known with the generic name of chitosan (Figure 4).

The chitosan family exhibits a high diversity in its members as a function of the degree (DA) and pattern of acetylation (PA) and the degree of polymerisation (DP). DA, PA, and DP are the key factors involved in the complex behaviour of chitosan in solution [33,34]. Chitosan dissolves in aqueous dilute acidic conditions at a pH lower than ~6.1–6.3, its intrinsic pK_0_ [35,36,37], due to the protonation of the amino groups and appearance of repulsive electrostatic forces. This also determines the stretching of the chains, showing both rigid [38,39] or semiflexible [40,41] conformation as a function of the environmental properties as well as of intrinsic features of these biopolymers. As a result, chitosan is a polycationic biopolymer in water solution.

Chitosans are biocompatible and biodegradable polymers that have antimicrobial activity and the ability to promote wound healing, among other interesting properties in the biomedical field. Another important bioactivity of (chitosan) CS is that it enhances the paracellular transport of active molecules [42,43]. This contributes to increasing the permeability of the payload of interest across the epithelial barrier [44,45]. Chitosan binds to integrins α_v_β_3_, which are in the membrane of intestinal epithelial cells. These integrins activate a signal route that involves focal adhesion kinase (FAK) and Src tyrosine kinases, inducing the degradation of the structural proteins of tight junctions in intestinal epithelial cells [45]. This activation mechanism induces the reversible opening of the tight junctions, thus enhancing the permeability of the drug while enhancing its systemic absorption. Another key role of CS is to protect active biomolecules which are transported in the polymeric structure. This fact increases the bioavailability of drugs and increases the probability that they reach their target sites.

There is a lot of interest in the application of CS in the biomedical and pharmaceutical fields due to their potential applications, such as drug delivery agent, blood anticoagulant, tissue engineering and space fillings material, among others. However to date, the commercial use of CS-based treatments are limited to those promoting wound healing and to haemostasis [46,47]. Understanding mucoadhesion and the nature of the interactions at play between CS, mucin, and the mucus gel have gained traction, particularly with a view to the development of smart drug delivery systems [48].

## 3. Mucoadhesive Properties of Chitosan

**Figure 5:**
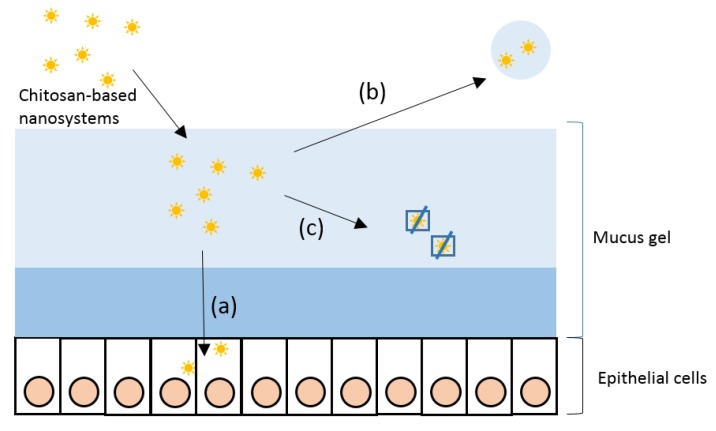
Possible scenarios in the interaction of chitosan-based nanosystems and mucins. Once the nanosystems achieve the mucus, they can go through the mucus and reach the epithelium (**a**), be shed with the mucus (**b**) or get trapped in the mucus gel (**c**).

The way in which chitosan and mucin interact determines the behaviour and fate of chitosan based nanosystems. Thus, the simplest postulation is that these types of biomaterials could be adsorbed at the mucus layer, shed with the mucus, or go through this layer and reach the cell surface. However, it could also occur that chitosan containing systems are covered by mucins, impeding their function (Figure 5). Thus, it is of great importance to study the mucoadhesive properties of chitosan and chitosan-based nanomaterials. To this end, different biophysical techniques can be used, such as viscosimetry, small-deformation rheology, microrheology, turbidimetry measurements, ζ-potential measurements, dynamic light scattering, isothermal titration calorimetry, fluorescence quenching studies, atomic force microscopy, confocal laser scanning microscopy, and transmission electron microscopy (TEM) [13,22,49].

**Figure 6:**
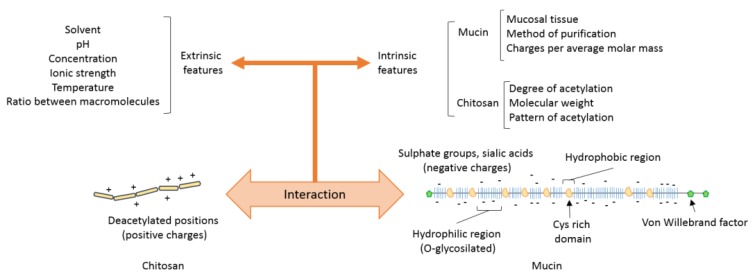
Intrinsic and extrinsic features that alter the interactions between chitosan and mucin.

Due to the great variability in the composition and structure of chitosan and mucin, there have been wealth of studies addressing the interaction between these macromolecules in which different raw materials have been used and often the obtained results are contradictory. Concerning mucins, since their structure is essentially the same in both types of mucins—secreted and cell-associated—the behaviour of secreted mucins is expected to be shared by those cell-associated mucins. Therefore, the experimental works are typically developed with secreted mucins, which may be typically obtained either as a commercial research reagent (e.g., from Sigma-Aldrich, Darmstadt, Germany) from various sources (e.g., pig stomach, bovine submaxillary cavity) and degrees of purity or else purified directly from excised mucosal tissues. In the first case, the composition is subject to high batch-to-batch variability and purity. In the second, the variability is given by both the type of mucosal tissue and the method of extraction. In regards to chitosan, the variability is given by the degree of acetylation (DA), molecular weight, and pattern of acetylation, whether neutral or salt form. Also, the properties of both are greatly affected by the solvent, temperature, and concentration (Figure 6). Overall, relatively little attention has been given to the understanding of the underlying mechanisms of the interactions between chitosans and mucins, and thus more systematic studies are needed using different type of chitosans and mucins.

## 4. Main Forces Behind the Interaction Between Chitosan and Mucin

Interactions between chitosan and mucins depend on several intrinsic factors (those associated directly with the nature of the polymers, e.g., molecular weight, surface charge, conformation) but are also highly dependent on the external or environmental factors where the interaction takes place (Figure 6). Some external factors have a direct influence on certain intrinsic factors and therefore also modulate the interaction exhibited.

Concentration is a key factor to be considered when studying the interaction between mucin and chitosan. With regard to chitosan, above the so-called critical concentration (c*), the chitosan chains overlap and form entangled networks [50,51]. Therefore, the chitosan chains will not be free to interact with glycoproteins of mucins. In the case of mucins, due to their physiological functions and structure, at high concentrations they are also known to form entangled and gel networks [10]. The interaction is dependent on the ratio between both macromolecules in solution. However, to estimate the number of charges per average molar residue, it is necessary to first know the composition of the constituent residues of the polymer or macromolecule. In the case of chitosan, this is readily available from the value of DA. However, this is not the case for mucin, given the complexity of its composition. Therefore, to express the composition of CS-mucin complexes the mass ratio is often used instead of the molar ratio.

Another important factor is the pH of the media. Chitosan has, in general, a pK_0_ around 6.1, therefore at pH lower than the pK_0_ the amine group in the *N*-acetyl-glucosamine units will be protonated, leading to electrostatic repulsion forces between them and therefore favouring a more extended conformation of the molecule. When the pH is equal or higher than the pK_0_, repulsion force action ceases and the chains get retracted and insoluble. Mucins, on the other hand, are glycoproteins with pI between 2 and 3 [10], therefore in solutions where pH > pI these are charged negatively, promoting electrostatic repulsion forces between them and giving rise to a more extended conformation of the chain [4,52]. Electrostatic complexation (the interaction between mucins and chitosan) is therefore likely to be favoured at a pH between 2.4 and 6.3.

Chitosan and mucin in aqueous solutions are known to interact predominantly electrostatically, yielding protein–polysaccharide complexes. Meng-Lund [53] found that interactions between chitosan and mucins occur in a two-step entropy driven process, which is favoured at a pH of 5.2, where interactions between molecules are stronger according to the apparent association constants (K_app_ (1st step) = ~1.75 × 10^6^ M^−1^ and K_app_ (2nd step) = ~10^6^ M^−1^) compared to pH = 6.3 (K_app_ (1st step) = ~5 × 10^5^ M^−1^ and K_app_ (2nd step) = ~2.5 × 10^5^ M^−1^). The work force of adhesion in each case was 0.3–0.5 mN and 0.2 mN, respectively (Table 1).

The interaction between CS and mucin is also affected by the chain flexibility of CS, which depends on the DA of the chain: the higher the DA, the more flexible the chain, due to the absence of electrostatic repulsion forces on the surface of the chain. Nevertheless, this behaviour is not always observed. According to the results from Menchichi et al. [22], CS with a chain length smaller than ~360 to ~730 residues shows a semirigid conformation, regardless of the DA of the chain (Table 1). In another work from our group [48], the intrinsic viscosity of the chitosan was shown to vary as a function of the ionic strength. A parameter termed degree of coil contraction was defined as the intrinsic viscosity of the polymer in water divided by the intrinsic viscosity of the polymer in NaCl to analyse the changes in the conformation of the polymer. The results indicated that the degree of coil contraction varies with the CS DA: HMC30 < HDP 56 < HDP 1 > HDP 11 = HDP 27 = HMC 15 (for details, see Table 1 and [48]).

The ionic strength of the media influences the interaction between both species. At a high salt concentration, the condensation of ions in the solution around opposite charges on the surface of the macromolecules screens electrostatic forces and the macromolecules behave as neutral species. This means that the chitosan and mucin become effectively uncharged, and consequently the contribution of electrostatic interactions is offset [50,51]. Menchichi et al. also studied the interaction between porcine gastric mucin (PGM) and six different types of CS, namely four biomedical grade CS with DA between 1.6 and 56 and Mw in the range of 123 to 265 kDa, and two pharmaceutical grade CS with DA equal to 32.4 or 14.8 and Mw in the range of 17–27.5 kDa. When suspensions of CS and PGM—both with a relative viscosity ~2.0—were mixed, a reduction in the relative viscosity of the resulting suspensions was recorded. This was taken as evidence of the heterotypic interaction between both polymers and was found to be dependent on the PGM weight ratio in suspension, the DA and Mw of CS, and the ionic strength of the environment. Of note, the reduction on the relative viscosity was higher in pure water than in 0.1 M NaCl, consistent with the prominent role of electrostatic interactions at play. Regarding the role of CS’s Mw, the reduction in the viscosity for low molecular weight CS was always lower in comparison with other CS [22]. Accordingly, the stoichiometric interaction between CS and PGM depends on the DA of the polyelectrolyte. These studies led to identifying the ‘stoichiometric point’ as the PGM weight content in which the relative viscosity of the solution had the minimum value and the ζ-potential was zero. Interestingly, these studies revealed that, unlike what happens with CS, if more PGM is added to the solution it remains free in the suspension [22]. It is noteworthy that, unlike the results from Meng-Lund [53], the Isothermal titration calorimetry (ITC) studies carried out by Menchichi et al. indicated that the CS–PGM interaction at pH 4.5 is a one-step enthalpy-driven process. This work was carried out at a lower pH, namely 4.5, with low molecular weight chitosan and a different type of mucin (Table 1). All these differences could be the reasons for the different results. In the work from Menchichi et al., the PGM showed a slightly higher affinity towards CS of intermediate DA (DA = 30%), where the K_app_ was 4.58 (±1.51) × 10^4^ M, than towards CS of low DA (DA 15%), whose K_app_ was 2.62 (±1.33) × 10^4^ M [22]. These results reveal a lower affinity between CS and PGM than that reported by Meng-Lund. The heterogeneity in the results obtained reveals that further studies are needed to unravel the thermodynamic nature of the reaction between both macromolecular species. Experiments to find out if mucins behave differently as a function of their source of isolation or as a function of the way of purification are also needed.

The interaction between mucin and polyanions is reduced when increasing the ionic strength of the medium. However, the interaction of PGM and CS in conditions of increased ionic strength remains, and varies as a function of CS’s Mw [22,48]. The experimental works from Menchichi et al. confirmed that the electrostatic interaction is important but also point out that other mechanisms of interaction are implicated as well. The hydrophobic interactions between the hydrophobic domains in mucins and the acetyl groups in chitosan have been proposed.

Dedinaite et al. [54] studied the interaction between CS with DA 15.5 and Mw 150 kDa and bovine submaxilliary mucins (BSMG) (Table 1) by ellipsometry and atomic force microscopy (AFM). For that purpose, a suspension of mucin, 0.025 mg/mL in 30 mM NaNO_3_, was applied on a mica surface. As a result, the glycoprotein got attached, producing a soft compressible layer, the adsorbed mucin was 0.5 mg/m^2^. According to the results of this group, the repulsive forces found in the mucin layer are higher than expected for the electrical-double layer, and hence the involvement of a steric contribution was hypothesised. When a solution of CS (0.02 mg/mL in 30 mM NaNO_3_) was applied, this polyelectrolyte produced the compaction of the mucin layer, the reduction of repulsive forces on the surface of layer of mucin, and the formation of dense composites. Washing of the layer with sodium dodecyl sulphate (SDS) does not result in the removal of CS or mucins. Then, they applied another layer of mucin and CS, which is considered as a cycle, and again, the layer formed resisted the washing. However, the repetition of another cycle, the third, yielded a structure which did not resist the washing and some content was removed when applying the SDS solution. The authors identified CS as the element that prevents the loss of mucin from the layers, since when the layer is only composed of mucin, if a solution of SDS is applied, nearly 80% of mucin is removed [54].

In another study using AFM, the interaction between PGM isolated from the stomach of pigs and CS of DA 1% and Mw 162 kDa or DA 49% and Mw 250 kDa was analysed. In this work, PGM (0.05 mg/mL) was immobilised on the mica surface, and a solution of CS (0.3 mg/mL) was used to modify the tip of the cantilever. According to the results, the interaction between PGM and CS was dependent on the DA of CS and on the solvent properties (Table 1). CS (DA 49%, Mw 250 kDa) showed an increase in the number of events of interaction with PGM when lowering the pH from 6.9 to 5.5, namely from 16.5% to 41.8%, respectively. The forces required for dissociation of PGM and CS were also measured. These forces rounded 0.2 nN, which were significantly lower than those referred to covalent bonds, which are 1–3 nN [55]. Accordingly, PGM and CS were proposed to interact due to electrostatic interactions, with hydrogen bonds and hydrophobic interactions playing an important role as well. The fact that CS with higher DA showed a higher number of interactions reveals the importance of the hydrophobic interactions between these macromolecules [55]. Another critical factor to take into account in the studies with AFM is the dilution of the sample, uncovered by the results from Li et al. (2010), who studied the interaction forces between CS (DA 15% and Mw 150 kDa) and a film of mucin (commercially available sample from stomach from Wako Chemical Inc., Osaka, Japan). In those situations, in which the tip of the cantilever was modified with a solution of CS at a concentration lower than 0.2%, repulsive forces were detected between both surfaces. On the contrary, when the concentration of the CS solution was higher than 0.2%, attractive forces were reported. These authors explain these results from the lack of hydrophilicity in the first case, which increased in the second scenario [56].

As a result of all the above, the interaction between both species is complex and difficult to understand and predict. It is important to point out that some research works describe that the interaction of chitosan and mucin in vivo alters the attachment of the biopolymer to the mucosal surfaces [11]. Thus, the study of the interactions between chitosan and mucin is of the utmost importance to advance in the knowledge of potential applications of these polymers.

## 5. Chitosan-Mucin Complexes: Characterisation and Mucoadhesion

Mucoadhesive hydrogels or nanocomposites obtained from the interaction between mucin and CS can have potential use for controlled and long-term delivery on the mucosal surfaces of biomolecules loaded inside them. At the same time, the absorption of such molecules and their bioavailability would be enhanced [57,58]. These systems could also be used in the development of treatments for increasing the lubrication of the mucosal surfaces in treatments for pathologies such as ulcerous colitis, which is related to colon cancer. Below are some works which studied the complexes obtained from the interactions between CS and different types of mucins.

Deacon et al. (2000) studied nanocomposites composed of PGM, isolated by them, and a glutamate salt of CS with DA 11% (Table 2). The results indicated that the PGM and chitosan molecules were easily distinguishable. The length of the PGM chains was 0.5 to 4.0 µm with a constant diameter of ca. 16 nm. In the case of the CS chains, their length was equal to 0.70 ± 0.27 µm, with a constant diameter of approximately 11 nm. The complexes composed of PGM–CS at 0.1 M seemed to be aggregates of the polymers. Although structures of different sizes can be seen, most of them showed a diameter equal to 0.70 ± 0.18 µm. The structure formed had no defined boundaries but filaments of PGM radiating outside the structure were formed, as was confirmed by the measurement of the diameter of the chains. When the ionic strength was increased, the complexes reduced their size and also a lower number of complexes were found [59].

Rossi et al. [60] used CS with DA equal to 84% and BSMG and PGM type III to prepare complexes. The CS and mucins were prepared at a relative viscosity from 1.0 to 1.5, and the complexes were prepared at a final CS:mucin ratio in the range of 1:0.5 to 1:20 in water and from 1:0.5 to 1:5 in 0.1 M HCl. The results showed that the stoichiometry of the interaction between CS and BSMG is a function of the pH of the medium. It is near 1:5 ratio in water and increases to 1:1 ratio in 0.1 M HCl. According to the authors, the change in the stoichiometry is likely due to the change in the conformation of the polymer, confirmed by the change in the intrinsic viscosity value between both solvents. The stoichiometry is also dependent on the type of mucin used. In the case of the PGM, the stoichiometry is close to 1:10 in water and 1:2 in 0.1 M HCl for the complexes composed of CS–PGM. These authors demonstrated that the presence of surplus mucin in the solution resulted in the precipitation of the complexes formed with the CS. In another work by the same research group [61], it was proven that there is a different rheological behaviour as a function of the CS:PGM ratios. A negative synergism was observed at a higher polymer ratio. In fact, the value of viscosity was minimum at a ratio CS:PGM 1:0.5 in water and 0.1 M HCl. Interestingly, a positive synergism was reported in conditions of a higher content of mucin CS:PGM 1:10 in water and 1:5 in 0.1 M HCl.

Complexes composed of CS with DA equal to 19% and Mw 113 kDa and PGM type II (Table 2) were synthesised by Silva et al. [11]. These complexes were distributed in two populations of micrometric (2795 ± 247 nm) and nanometric (478 ± 112 nm) size. In this work, the interaction of the CS, the PGM, and the complexes composed of both macromolecules with Langmuir–Blodgett films of dimyristoilphosphatidic acid was analysed. The results indicated that the complexes formed interact with the LB films, either by electrostatic interactions through CS or by hydrophobic interactions via PGM. Besides this, the interaction between CS and PGM on the membrane is so favoured that if PGM is adsorbed on the membrane and CS is added, PGM can be removed by interaction with CS.

Menchicchi et al. [22], as discussed previously, studied the interaction between six different types of CS of varying DA and Mw and PGM type III (Table 2). Samples of CS and PGM were prepared with a relative viscosity of approximately 2.0. In the case of PGM, the pH was set to 4.5 and the solvent conditions were either water or 0.1 M NaCl solution. Then, mixtures of both polymers, CS and PGM, were prepared at varying mass fractions of mucin (*f*), *f* = 0.2, 0.4, 0.6, and 0.8. Their results showed that, as the content of PGM fraction in the samples increased, the hydrodynamic diameter of the complexes containing high Mw CS (DA 1–27%) decreased, while the complexes containing either low or high Mw CS (DA 56%) did not show any change in size at the different *f* values. In all cases, an increment in the size was reported in those conditions of minimum viscosity. Regarding the ζ potential with *f* for the complexes formed by the different CS, it was shown that the values at which neutral complexes are obtained decreases as the DA of CS increases.

Nikogeorgos et al. [13] prepared structures composed of CS (DA 80% and Mw 250 kDa) and PGM type III (Table 2) at different CS:PGM weight ratios. The results indicated that at pH 3.2, maintaining a constant concentration of the total polymer in suspension, the ζ potential of the complexes increased when the content of CS was increased, the same result was reported previously by Menchichi et al. [22]. The electroneutral complexes prepared by Nikogeorgos et al. were formed with a CS weight fraction equal to 0.33. This value is close to that obtained for complexes with a CS 56% DA at pH 4.5, synthesised by Menchichi et al., who reported a CS weight fraction equal to 0.3. This similarity, despite the different DA of the CS, may be due to the different pH values of the solutions (3.2 versus 4.5) used for each study. Concerning the size of the complexes, a decrease in the size of the final structures was found when CS was added to a solution of PGM at pH 3.2. It was seen that the higher the CS content, the lower the hydrodynamic size of the complexes. This behaviour is different from that reported by Menchichi et al. (2014) [22], who found that complexes composed of CS 56% DA and Mw similar to that used by Nikogeorgos et al. (2015) [13] in a weight fraction from 0 to 0.4 increased in size as the CS amount was increased. For higher CS weight fractions, the size of the complexes diminished as the amount of CS was increased. Due to this, at pH 3.2 the only charged groups in PGM are the sulphate groups, the interaction between polymers is achieved through the electrostatic and coulombic interactions between SO^‒^_4_ in PGM and NH^+^_4_ in CS. In the same work, the effect of the lubricity of these complexes on a membrane of polydimethylsiloxane (PDMS) was studied. According to the results, the higher the CS weight ratio of the complexes is, the lower the friction coefficient (µ). The interaction is dependent on the dynamic viscosity, since only the interaction with the complexes increases the lubricity, while the interaction with PGM or CS does not produce the same effect. Therefore, the interaction of the polymers to produce the complexes before applying to the membrane is crucial. According to the friction force microscopy results, the energy needed for breaking the interaction between the complexes and PDMS membrane is higher than that needed for breaking the interaction of either PGM or CS with the PDMS membrane. In fact, the higher the CS weight content is, the higher the energy required. According to the authors, the interaction between the complexes and PDMS membrane is a process driven by two properties: the net charge of the complexes and their size. Since the interaction is a diffusion process, the smaller the complexes are, the more likely the interaction with the PDMS membrane will be.

## 6. Mucoadhesion of Chitosan-Mucin Complexes

As discussed in the introduction, the mucoadhesion behaviour of the complexes composed by CS and mucin is important in order to determine the interaction of the complexes with the mucus.

Kootala et al. [62] used CS with a DA lower than 1% and different DPs, namely 8, 52, and 100, and consequently different Mw (Table 2), and mucin purified from porcine stomach by themselves and bovine submaxillary mucin glandule (BSMG) to study the interaction of the CS and mucins and to prepare complexes that were applied on HT29-MTX cell cultures, a cell line that produces mucus. Mucins were immobilised over layers of polyethylene glycol (PEG) to analyse the interaction with CS. The results revealed that the lower the DP is, the stronger observed interaction with the mucin, with both PGM and BSMG. In this work, it was proven that the immersion of a drop of a solution of CS at 5 mg/mL in a suspension of mucin isolated from porcine stomach resulted in the formation of a hydrogel composed of CS and mucin. The hydrogels synthesised in this way were analysed by stereomicroscopy, confocal laser scanning microscopy, and cryo-scanning electron microscopy (CryoSEM). These three microscopy techniques used in this work allowed us to visualize the hydrogels at different levels of magnification. The results revealed that CS with lower DP showed a homogeneous distribution of CS in the hydrogel, and the pore size of these structures was the lowest of the hydrogels synthesised. Moreover, the hydrogels prepared with CS with DP 52 showed a heterogeneous distribution of CS, increasing the distribution in the periphery of the structure. In the case of the hydrogel composed of DP 100, the CS was unable to go inside the structure formed and it was found in the periphery of the hydrogels. Despite the different disposition of CS on the hydrogels, the amount of CS attached to the mucin to form the hydrogels was similar in all cases—approximately 0.3 µg of CS per µg of mucin. The complexation of CS with mucins increased the efficacy of the barrier controlling the diffusion of particles through it, as can be deduced from the decrease in the diffusion of labelled particles through the hydrogels. The study of the effect of CS on the mucus layer in HT29-MTX cell culture revealed that the CS with DP= 8 diffused through all the layers, and the diffusion of small molecules (dextran and cholera toxin B) fluorescently labelled was not affected. On the contrary, the CS DP 52 and 100 did not diffuse inside the mucus layer, but formed a sheet on the mucus layer that impaired the diffusion of particles and small molecules inside the mucus layer.

Szymańska et al. [63] prepared different tablets, varying the content of CS with DA in the range of 15 to 25%. The mass ratio in the final formulation ranged from 10 to 40%. The results revealed that the formulation produced with 40% of CS yielded unstable tablets but those tablets, with a content of CS between 10 and 30%, were uniform and stable. The mucoadhesion of the tablets was studied on porcine vaginal mucus. The results showed that the mucoadhesion of the synthesised complexes was greater as the content of CS increased, indicating that a higher content in CS led to higher work of adhesion, force detachment, and longer residence time in the mucosa. Other complexes composed of CS with a DA of 10–25% and Mw of 70–150 kDa were prepared in another work. These complexes were used to load insulin. The resulting complexes showed a hydrodynamic size of 412 ± 7 nm and a positive ζ-potential (+36 ± 1 mV), and they were applied to a Caco-2 cell culture and to a co-culture of Caco-2 and HT29-MTX cells. Unlike HT29-MTX, Caco-2 cells do not produce mucus. The application of the insulin-loaded NPs increased the intracellular fluorescence in the Caco-2 cell culture, while the fluorescence in the cells from the co-culture was lower, probably due to the presence of mucus in the co-culture. Another effect of the presence of mucus was the different level of decrease in the cell viability produced by the complexes. The decrease was lower in the co-culture cell, the presence of mucus could be responsible for the protection of the cells [64].

## 7. Chitosan Cross-linked Structures

The method of preparation of the microspheres influences the final characteristics of the structures, as was proven in the work of Dhawan et al. [65] (2004). CS with an Mw of 600 kDa was used to prepare microparticles by different methods, such as thermal gelation, emulsification, and ionic gelation and cross-linking with Tripolyphosphate (TPP) or glutaraldehyde. The microspheres produced by thermal gelation (TCL) had the lowest ζ-potential, while the microparticles obtained as a result of the ionotropic gelation had the highest ζ-potential. In the case of the microparticles chemically cross-linked, the ζ-potential decreased when the content of the cross-linker in suspension was increased. The microparticles were immersed in a mucin suspension and the amount of mucin adsorbed to them was analysed. According to the results, the content of mucin adsorbed was greater as the ζ-potential of the complexes increased, except for the microparticles obtained through thermal gelation, the conformation of which should be disrupted during the process. In the case of the ex vivo studies, the adsorption of the microspheres in the small intestine of rats was analysed. The results indicate that the adsorption was dependent on the synthesis method of the microspheres. The adhesion was maximum for those microspheres obtained by ionic gelation. Also, the content of cross-linker was important in the case of chemically crosslinked microparticles, since the amount of CS adsorbed to the tissue was higher when decreasing the cross-linking level.

Feng et al. [57] produced nanoparticles composed of CS with DA 11% and Mw 10 kDa and carboxymethyl chitosan (CMCS) with DA 19% and Mw 12 kDa with 92% of substitution (Table 3). Two different methodologies were applied, namely ionotropic gelation with TPP (CS/CMCS/TPP) or with calcium (CS/CMCS/Ca^2+^). The resulting nanoparticles were loaded with doxorubicin hydrochloride (DOX, log P 1.27), which shows no attachment to the mucin. According to their results, when applying the loaded NPs, the content of DOX in the mucin was enhanced. The content of DOX in the mucin was 1.1–2.1 times higher when applied with CS/CMCS/Ca^2+^. The ex vivo mucoadhesion was also analysed, the results showed that the NPs that did not contain TPP had better adhesion. Again, the mucoadhesion can be described by two steps. First, the intimate contact and wetting of polymers took place. In the second step, physicochemical interactions yielded the consolidation and strengthen of the adhesion. In other research work developed with CS with DA lower than 15% and Mw 150 kDa (Table 3), cross-linked nanoparticles with a ratio TPP:CS equal to 37.5% showed a hydrodynamic size equal to ≈120 nm and a ζ-potential equal to ≈+30 mV when loaded with insulin. The study on mucus from jejunum of porcine intestine revealed that nearly 95% of the nanoparticles were retained in the mucus. Although the effective diffusivity in the mucus was low (0.2 µm^2^/s), as well as the mean squared displacement in 2 h (=4 µm^2^), the evaluation of the efficiency of the systems, developed through the evaluation of the permeation of the insulin through the intestine, was carried out. To this end, different segments of rat intestine (jejunum, ileum, and duodenum) were used. The results indicate that the permeation of the insulin shows a direct correlation with the mucus layer on the mucosa, with the highest correlation in the jejunum (mucus layer thickness = 123 ± 4 µm), followed by the duodenum (170 ± 38 µm), and the lowest correlation in the ileum (480 ± 47 µm). With this experiment, Zhang et al. [66] showed that not only the mucoadhesion is important to achieve the bioavailability of the drugs transported, but also that the ability of the structures to penetrated the mucus plays a key role in the permeation efficiency.

## 8. Conclusions

As discussed in this review, the molecular interaction mechanism between mucin and chitosan is complex [48]. It depends on the intrinsic properties of both species (mucin and CS) and also on the environmental conditions (solvent, pH, ionic strength, temperature) of the system in which both molecules interact. The interaction between the macromolecules occurs mainly due to electrostatic attraction, hydrogen bonding, and hydrophobic interactions [13,67]. The specific domains implicated in these interactions have not been completelyuncovered, though it is known that the interactions are enhanced with the molecular weight and the degree of acetylation of the biopolymer [22,48].

Due to the different properties and behavior of both chitosan and mucin as well as their behaviour as a function of intrinsic and extrinsic characteristics, it is difficult to find the stoichiometric ratio at which the macromolecules will interact to produce complexes with potential interest for drug delivery.

Works to date on mucoadhesion of chitosan have revealed that the degree of polymerisation has an inverse relationship with the bioavailability of drugs loaded in the complexes. As it was discussed here, the higher the DP, the less homogeneously distributed the complexes in the mucosa are and the lower the bioavailability of the drugs is. The selection of chitosan as a function of its DA and DP is essential for the development of drug delivery systems which enable the engineering of stable complexes with tailored properties such as high levels of mucoadhesion that enhance the bioavailability of the payload.

## Figures and Tables

**Table 1:** Interaction between chitosan and mucins.

Chitosan	DA (%)	Mw (kDa)	C Chitosan (mg/mL)	Mucin	C Mucin (mg/mL)	Solvent	T (°C)	Method	Ref.
Chitopharm L Chitopharm M Chitopharm S from Cognis SmbH	N/S	500–5000 100–2000 50–1000	0.6	Porcine gastric mucin type II with bound sialic acids 1% (Sigma-Aldrich)	0.5	0.0125 M acetic acid at pH 5.2 or 0.016 M 2-(*N*-morpholino)ethanesulfonic acid at pH 6.3	25	Complex coacervation Isothermal titration calorimetry	[53]
Chitosan containing ≤1% insoluble matterfrom Fluka	15.5	150	0.02	Bovine submaxiliary mucins (BSMG) from Sigma	0.025	20 mM NaNO3. The pH was 7.5 for mucin and 4 for chitosan	N/S	Surface force measurements Atomic force microscopy	[54]
Chitosanfrom Katakurachikkarin Co Ltd.	15	150	Attached to the cantilever	Mucin form stomach from Wako Chemical Inc.	Dry film	No solvent, the AFM worked in air conditions	25	Atomic force microscopy	[56]
Two pharmaceutical grade CS HMC 15 HMC 30 from Heppe Medical Chitosan (HMC) GmbH	14.8 32.4	27.5 17	10	Porcine gastric mucin type III (Sigma-Aldrich)	5	20 mM acetate buffer, pH 4.5	25	Microviscosimetry Isothermal titration calorimetry	[22]
Four biomedical grade HDP 1 HDP 11 HDP 27 HDP 56 from Mathani chitosan Put. Ptd.	1.6 11 27.5 56	124 122 143 266	HMCs: 3–4 HDPs: 0.3–0.9		8	MilliQ water and 0.1 M NaCl, both of them at pH 4.5	37	Vicosity measurements	[48]
Two types of chitosan	1 49	162 250	Cantilever modified with chitosan	Mucin samples from cardia and fundus of pigs	Film, mucin linked to a mica surface	25 mM Hepes buffer, 150 mM NaCl, pH 6.9 and 25 mM acetate buffer, 150 mM NaCl, pH 5.5	25	Atomic force microscopy	[55]

N/S: not specified.

**Table 2:** Nanostructures composed of chitosan and mucins.

Chitosan	DA (%)	Mw (kDa)	C Chitosan (mg/mL)	Mucin	C Mucin (mg/mL)	Solvent	T (°C)	Method	Ref.
Glutamate salt of chitosan from Pronova Ltd. Drammen,	11	N/S	N/S	Porcine gastric mucin (PGM) isolated by them	N/S	0.1 M sodium acetate buffer, pH 4.5, 0.1 M NaCl. The ionic strength was increased up to 0.2 and 0.3 M	N/S	Complexes attached to mica surfaces and analised by atomic force microscopy performed in air	[59]
Chitosan HCl, high viscosity grade. Seacure^®^ CL 313 Pronova Biopolymers a.s.	84	N/S	0.02–0.08 in H_2_O, pH 4.6 0.2–0.8 in 0.1 M HCl, pH 1	Purified mucin from bovine submaxillary glands (type I) BSMG and Partially purified mucin Pig stomach type III (sigma)	BSMG: 0.1–0.6 in H_2_O, pH 6.8 or 0.4–1.6 in 0.1 M HCl, pH 1 PGM: 0.4–1.6 in H_2_O (pH 4.7–5.1) or 0.1 M HCl (pH 1)	0.1 M HCl or distilled water	N/S	Viscosity measurements	[60]
Chitosan HCl (high viscosity grade) (HCS) Seacure^®^ CL 313. Pronova Biopolymer	N/S	N/S	5–40 in H_2_O and 15–50 in 0.1 M HCl	Partially purified mucin from pig stomach (Sigma)	7.5–400 in H_2_O and 10–500 in 0.1 M HCl	0.1 M HCl or distilled water	N/S	Viscosity measurements and tensile stress test	[61]
Chitosan from Galena Farmacêutica	19	113	0.1	Porcine gastric mucin type II with bound sialic acids 1% (average Mw 29 MDa)	0.1	Langmuir monolayer	N/S	Fourier transform infrared spectroscopy (FTIR) and quartz crystal microbalance (QCM)	[11]
Chitosan MMW with a viscosity near 1% solution in 1% acetic acid equals to 200 cP from Sigma Aldrich	15–25	N/S	Tablets containing chitosan	Porcine vaginal mucosa from large white pigs weighing ~200 kg	Porcine vaginal mucosa or 10% mucin gel was absorbed on a cellulose fiber	0.08 M acetic buffer, pH 4.5	37	Measurement of detachment force and work of adhesion by using texture analyser	[63]
Two pharmaceutical grade CS HMC 15 HMC 30 from Heppe Medical Chitosan (HMC) GmbH	14.8 32.4	27.5 17	10	Porcine gastric mucin type III (Sigma)	5	20 mM acetate buffer, pH 4.5	25	Microviscosimetry Isothermal titration calorimetry	[22]
Four biomedical grade HDP 1 HDP 11 HDP 27 HDP 56 from Mathani Chitosan Put. Ptd	1.6 11 27.5 56	124 122 143 266							
Chitosan Batch 1001135895, from sigma	80	250	0.1	Porcine gastric mucin type III (Sigma)	0.1	1:1 (v/v) 0.01 M phosphate buffer saline (pH 7.4) and 0.01 M HCl. The final pH was 3.2	RT	ζ-potential, dynamic light scattering, optical waveguide light-mode spectroscopy, tribometry and tribopair	[13]
Three chitosans with DP 8 DP 52 DP 100 from Mahtani chitosan Ltd. India	<1%	1.3 8.4 16.1	5	Mucin purified from porcine stomachs. The mucins isolated were MUC5A and MUC5B Commercial bovine submaxillary mucins (BSM) from Sigma	Immobilized on a disc or in solution at 10 mg/mL	Acidified PBS, pH 5.5	25	quartz crystal microbalance with dissipation (QCM-D) and microscopy	[62]
Ultra-pure chitosan chloride (CS protasan UP CL 113) from Nova Matrix	10–25	70–150	1	RevHT29MTX cell line Adenocarcinoma cell line Caco-2 from ATCC	In cell culture	Water and DMEM	37	Dynamic light scattering, ζ-potential, microscopy	[64]

N/S: not specified, RT: room temperature.

**Table 3:** Chitosan (CS) nanoparticles crosslinked.

Chitosan	DA (%)	Mw (kDa)	Method of Crosslinking	Mucin	C Mucin (mg/mL)	Solvent	T (°C)	Method	Ref.
Chitosan with a viscosity at 1% in acetic acid 1% at 20 degrees equals to 400 mPa/s from Fluka	N/S	600	Thermal Glutaraldehyde Tripolyphosphate, Emulsification Ionotropic gelation	Mucin type III partially purified from porcine stomach, bound sialic acids ~1% (Sigma)	0.025–0.5	Milli-Q water	RT	Mucus glycoprotein assay	[65]
Chitosan from Jinan Hai debei marine bioengineering co. Ltd.	≤15	150	Ionotropic gelation	Mucus from jejune segments of porcine intestine	Mucus gel	Kreb’s-Ringer buffer	37	Multi particle tracking and confocal studies	[66]
CS from Biotech Co.	11	10	Ionic gelation plus crosslinking with tripolyphosphate or calcium	N/S	0.5–2.5	Oxygenated Kreb’s-Ringer buffer	37	Mucus glycoprotein assay	[57]
Carboxymethyl chitosan of 92% of substitution synthesized and characterized by authors	19	12							

N/S: not specified, RT: room temperature.
